# Structural, Spectroscopic, Thermal, and Magnetic Properties of a New Dinuclear Copper Coordination Compound with Tiglic Acid

**DOI:** 10.3390/ma14092148

**Published:** 2021-04-23

**Authors:** Marcin Świątkowski, Suneel Lanka, Agnieszka Czylkowska, Katarzyna Gas, Maciej Sawicki

**Affiliations:** 1 Institute of General and Ecological Chemistry, Lodz University of Technology, Zeromskiego 116, PL-90924 Lodz, Poland; suneel.lanka@dokt.p.lodz.pl (S.L.); agnieszka.czylkowska@p.lodz.pl (A.C.); 2 Institute of Physics, Polish Academy of Sciences, Aleja Lotnikow 32/46, PL-02668 Warsaw, Poland; kgas@ifpan.edu.pl (K.G.); mikes@ifpan.edu.pl (M.S.)

**Keywords:** coordination compound, copper, tiglic acid, crystal structure, IR spectroscopy, thermal analysis, magnetic properties

## Abstract

The first coordination compound of copper and tiglic acid named tetrakis(μ-tiglato)bis(tiglic acid)dicopper(II) was synthesized and crystallized from water solution. Its structure was determined and analyzed based on X-ray diffraction measurement. The paddle-wheel coordination system of the investigated compound was compared with other similar copper structures known in the literature. The Hirshfeld analysis was used for the detailed analysis of intermolecular interaction. The new compound was also characterized in terms of infrared absorption, thermal, and magnetic properties. The antiferromagnetic coupling of copper ions was found.

## 1. Introduction

**Figure 1:**
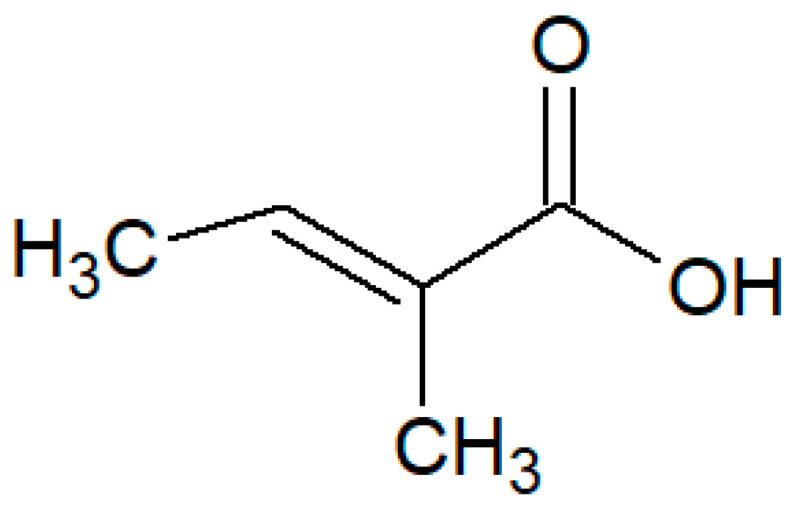
The chemical formula of tiglic acid.

More commonly known as tiglic acid, (*2E*)-2-Methylbut-2-enoic acid is one of the simplest unsaturated monocarboxylic acids (Figure 1). It is a volatile, crystalline solid with a distinctive, sweet odor. Tiglic acid naturally occurs in croton oil. It can also be found in the secretions of certain species of beetles [1,2]. Along with angelic acid, it forms a pair of cis–trans isomers. 

Unsaturated organic acids are important compounds considering their industry applications. Their esters are widely used in the food, cosmetic, and pharmaceutical industries [3]. Tiglic acid is not an exception—along with its derivatives, it is an important flavoring agent and fragrance additive. Tiglic acid can be used in the processes of manufacturing rum, caramel, bread, and fruit essences [4]. Its derivatives also exhibit potential anti-inflammatory [5] and antiproliferative activity [6]. One of the biggest areas of interest concerning tiglic acid is its biosynthesis—naturally occurring flavors and fragrances synthesized using enzymes can be labeled as “natural” [3,7]. The coordination chemistry of tiglic acid has not been widely explored to date [8,9,10,11]. In the Cambridge Structural Database (CSD) [12], there are only 15 compounds whose structures contain such acid or its anion; thus, every new research effort in this field provides important knowledge and fills the existing literature gap. 

The carboxylic acids exhibit a tendency to form dinuclear coordination compounds with copper. Such compounds possess the paddle-wheel structure in which two copper cations are bridged by four carboxylate anions. Most often, the paddle-wheel structure is completed by two terminal axial ligands. Research on dinuclear copper compounds has been conducted since the 1950s [13,14,15], and they are still of great interest due to i.a. magnetic properties [16,17,18,19,20,21,22]. The spin exchange parameter (−2 *J*) for such compounds is most often in the range 200–600 cm^−1^, which means antiferromagnetic coupling [23,24,25]. The value of this parameter is not only dependent on Cu•••Cu distance but also on other structural parameters, e.g., lengths of Cu-L(axial) bonds, lengths of Cu-O-C-O-Cu bridges, O-Cu-O, and Cu-Cu-O angles as well as on the electronic properties of ligands [23,24,25]. 

This work presents the synthesis, crystal structure, and study of spectroscopic, thermal, and magnetic properties of the new coordination compound of copper and tiglic acid tetrakis(μ-tiglato)bis(tiglic acid)dicopper(II).

## 2. Materials and Methods

### 2.1. Synthesis of [Cu_2_(tig)_4_(tigH)_2_]

Copper(II) carbonate hydroxide (15.9 g, 0.0721 mol, POCh, Gliwice, Poland) and tiglic acid (28.0 g, 0.2800 mol, Sigma-Aldrich, Darmstadt, Germany) were placed in a round-bottom flask with 250 cm^3^ of water. The reaction mixture was heated under a reflux condenser for 6 h. Then, the unreacted copper(II) carbonate hydroxide was filtered off and the clear solution was left to crystallization at room temperature. Green single crystals of [Cu_2_(tig)_4_(tigH)_2_] were obtained after several weeks. They were filtered and dried in the air. The yield of the synthesis calculated in relation to tiglic acid (substrate used in deficiency) was 56% (resulting mass 18.9 g).

### 2.2. Crystal Structure Determination

X-ray diffraction data of [Cu_2_(tig)_4_(tigH)_2_] were collected on a Rigaku Synergy Dualflex automatic diffractometer (Rigaku Corporation, Tokyo, Japan) equipped with Pilatus 300 K detector and microfocus sealed PhotonJet X-ray tubes, with shutterless ω scan mode. Lorentz, polarization, and empirical absorption (using spherical harmonics, implemented in SCALE3 ABSPACK scaling algorithm) corrections were applied during the data reduction. The structure was solved with a dual-space algorithm (SHELXT [26]). All nonhydrogen atoms were refined anisotropically using a full-matrix, least-squares technique on *F*^2^ (SHELXL [27]). All hydrogen atoms were refined using the “riding” model. Isotropic displacement factors of hydrogen atoms were equal to 1.2 times the value of an equivalent displacement factor of parent methine carbon atoms, and 1.5 times of parent hydroxyl oxygen and methyl carbon atoms. Structural visualizations were made in Mercury CSD 4.3.0 (Cambridge Crystallography Data Centre, Cambridge, UK) [28]. Hirshfeld surface maps and the fingerprint plots were generated using Crystal Explorer 17.5 (University of Western Australia, Nedlands 6009, Australia) [29] Details concerning crystal data and refinement are given in Table 1. 

CCDC 2045148 contains the supplementary crystallographic data for this paper. These data can be obtained free of charge via http://www.ccdc.cam.ac.uk/conts/retrieving.html (or from the CCDC, 12 Union Road, Cambridge CB2 1EZ, UK; Fax: +44-1223-336033; E-mail: deposit@ccdc.cam.ac.uk).

### 2.3. Magnetic Measurements

Magnetic studies were performed in a commercial superconducting quantum interference device (SQUID) magnetometer MPMS XL5 of Quantum Design. To facilitate adequately sensitive SQUID measurements of powdered substances, gelatin capsules or other unreliable containers were not used. Instead, the powder material was stabilized with a strongly ethanol-diluted GE-varnish [30]. GE is a well-known low temperature bonding agent particularly handy for such studies as it introduces a marginally weak magnetic flux. Subsequently, the wet mixture was transferred onto a 5 × 4 × 0.15 mm^3^ piece of previously well magnetically characterized silicon (Si). The Si plate provides a solid support and eases the handling of the specimen. After evaporation of ethanol, the GE-stabilized powder and the Si plate form a robust structure, which survived perfectly intact the mounting in the magnetometer and the whole magnetic *H* field and temperature *T* cycling applied during the measurements. The relatively weak magnetic signal of the Si base plate was adequately removed from the results, yielding the magnetic response of the investigated [Cu_2_(tig)_4_(tigH)_2_]. For the measurements, the whole structure was affixed at the center of about a 20 cm long silicon strip using the same dilute GE varnish. The Si strip takes the role of the sample holder assuring the adequate sample position with respect to the SQUID pick-up coils, without introducing any detectable signal. All the measurements, data reduction, and final magnetic moment determination were performed following strictly the already described procedures adequate for high sensitivity studies of a sample of minute magnetic signals [31].

### 2.4. Other Measurements

The elemental analysis of C, H, and O was carried out using a Vario EL III CHNOS Elemental Analyzer (Elementar, Langenselbold, Germany). The Cu content was determined based on edta titration in the presence of 1-(2-pyridylazo)-2-naphtol as an indicator [32]. Analysis for the studied compound [determined/theoretical (%)]: C 48.9/49.8; H 6.0/6.1; O 27.1/26.5; Cu 16.9/17.6. The FT-IR spectra were recorded on a Jasco FT/IR 6200 spectrophotometer (JASCO, Easton, MD, USA), in the form of KBr pellets, in the spectral range 4000–400 cm^−1^, with resolution 1 cm^−1^. The thermal decompositions were carried out with a Netzsch STA 449 F1 Jupiter thermoanalyzer (Netzsch-Geratebau GmbH, Selb, Germany) coupled with a Netzsch Aeolos Quadro QMS 403 mass spectrometer (Netzsch-Geratebau GmbH, Selb, Germany). Samples were heated in Al_2_O_3_ crucibles, in the temperature range 35–1000 °C, with the heating rate 10 °C/min in synthetic air (80% N_2_, 20% O_2_).

## 3. Results and Discussion

### 3.1. Structural Analysis

**Figure 2:**
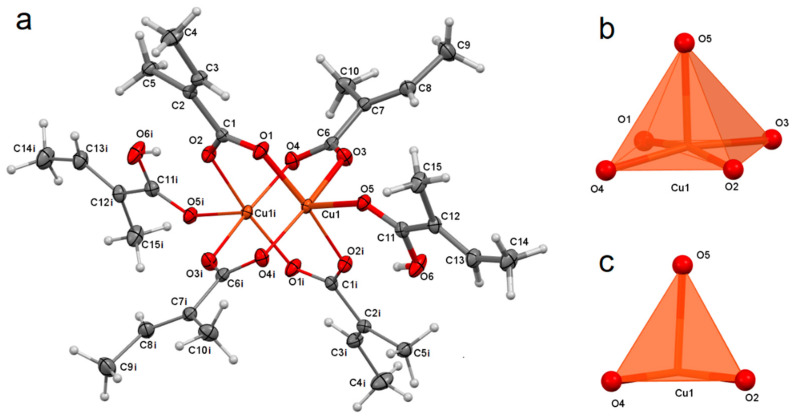
Molecular structure of [Cu_2_(tig)_4_(tigH)_2_], with atom numbering scheme, plotted with 50% probability of displacement ellipsoids of nonhydrogen atoms. Hydrogen atoms are plotted as spheres of arbitrary radii. The symmetry generated atoms are indicated by i letter [symmetry code: −x + 1, −y + 1, −z + 1] (**a**). Coordination polyhedron of [Cu_2_(tig)_4_(tigH)_2_], general view (**b**), view along tetragonal base (**c**).

The studied compound tetrakis(μ-tiglato)bis(tiglic acid)dicopper(II) is the first coordination compound of copper with tiglate anion, the structure of which was determined [12]. This is a dinuclear compound, whose two copper cations are bridged by four tiglate anions with syn–syn mode, forming a paddle-wheel structure (Figure 2a) [33]. The coordination sphere of the central atom is completed by monodentate tiglic acid coordinating by carbonyl oxygen. The coordination polyhedron adopts the geometry of a tetragonal pyramid (Figure 2b,c), in which the equatorial positions are occupied by oxygen atoms of anions and the axial position by carbonyl oxygen of acid. The presence of the inversion center (special position *b* of *P*2_1_/*c* space group) in the middle between copper cations makes that the one-half of the compound [Cu(tig)_2_(tigH)] is equivalent to the second one. The bond valence sum of Cu1 is 1.976 (Table 2) [34,35,36,37], and it is close to the formal charge 2+ of copper, which proves that Cu•••Cu interaction has a nonbonding character [38]. 

**Figure 3:**
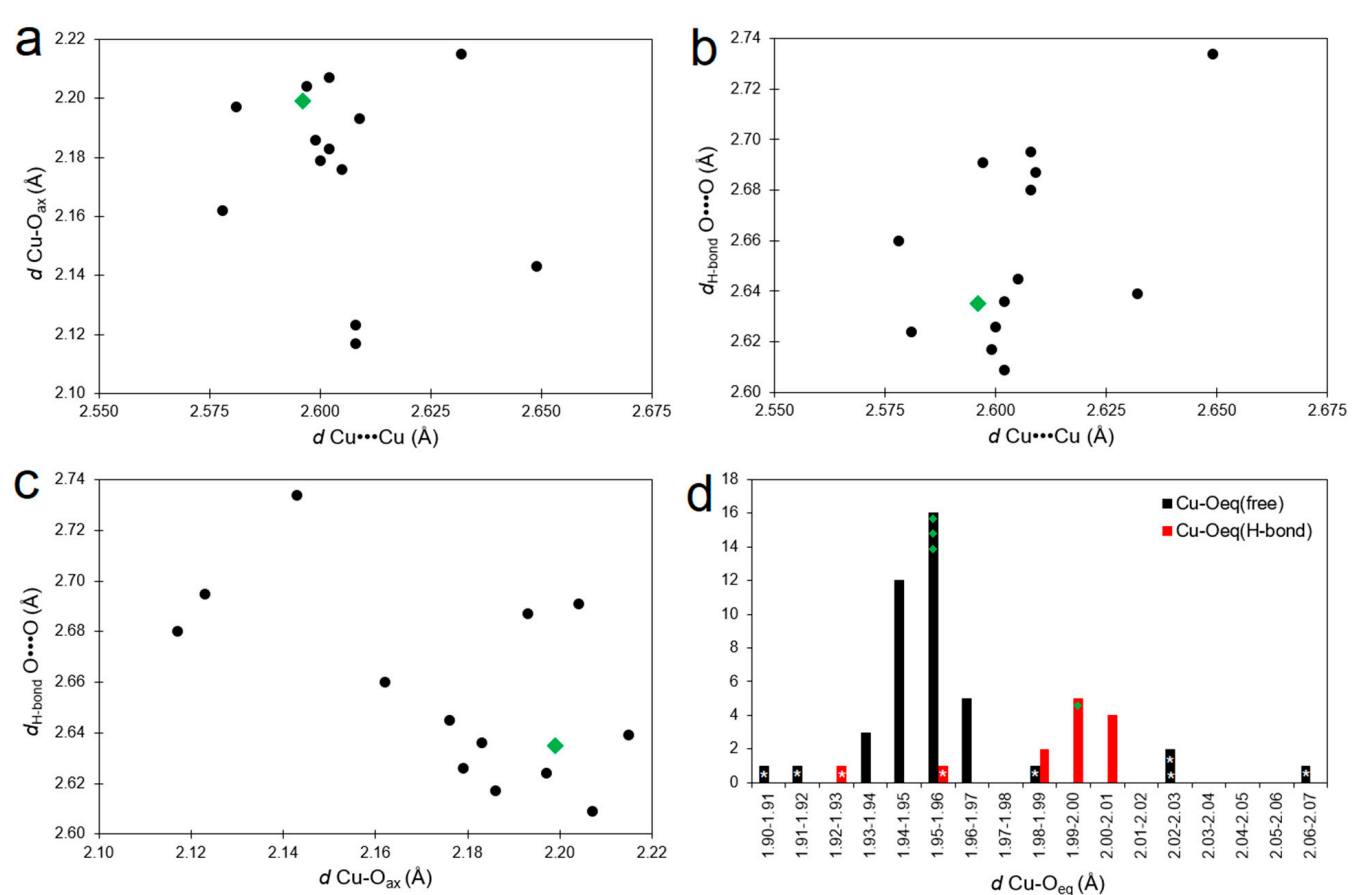
Scatterplots (**a**–**c**) and histogram (**d**) presenting relationships between selected structural parameters in [Cu_2_(A)_4_(HA)_2_] compound group (CSD refcodes of included structures: ACACCV10, FAKRUE, GUGROP, KALQET, KIJKUJ, KUBHOE01, LATXEL, MODCOY, QIXGOU, ROZLUN, SUGHIL01, WEVWEZ). Green color indicates data of [Cu_2_(tig)_4_(tigH)_2_]. White stars indicate data of [Cu_2_(triphenylacetate)_4_(triphenylacetic acid)_2_] (CSD refcode: ROLZUN). For compounds, in which both copper cations are symmetry-dependent, one data set is presented. Two data sets are included only for ROZLUN, whose copper cations are not equivalent.

The structural features of the studied compound were compared with 12 known dicopper compounds with the formula [Cu_2_(A)_4_(HA)_2_] (where: HA is a carboxylic acid and A is its anion) [12]. It was reported that Cu•••Cu distance depends on an axial ligand [39]. More nucleophilic axial ligands lead to a lengthening of dicopper distance as a result of the formation of a stronger Cu-L(axial) bond. Taking into account only carboxylic acids as the axial ligands allows estimation of the strength of Cu-O(axial) bonds based on their length. The comparison of Cu•••Cu distance with Cu-O(axial) length for [Cu_2_(A)_4_(HA)_2_] compounds shows no significant relationship (Figure 3a). In the studied compound, both mentioned structural parameters (Table 2) are within standard deviations of the mean values (2.61 ± 0.02 Å and 2.17 ± 0.03 Å, respectively) calculated for the group of [Cu_2_(A)_4_(HA)_2_] compounds. The lack of correlation between mentioned parameters means that differences in strength of acids resulting from different substituents bonded to a carboxylic group do not influence the dicopper distance. It can be a consequence of the intramolecular hydrogen bond formed between the hydroxyl group of acid and the oxygen of one bridging anion. The formation of S(6) hydrogen-bonded ring introduces some strains to the structure, which can affect a disruption of the correlation between the above-discussed structural parameters. While the direct influence of h-bond strength on the dicopper distance is not observed (Figure 3b), there is an inverse relationship between h-bond O(donor)•••O(acceptor) distance and the Cu-O(axial) bond length (Figure 3c). It means that an increase in strength of the intramolecular h-bond causes the weakening of the Cu-O(axial) bond. This h-bond in the studied compound is one of the strongest in the [Cu_2_(A)_4_(HA)_2_] group. It is a consequence of coupling between the carboxylate group and the double bond, which increases the nucleophilic character of the h-bond acceptor. Moreover, the involvement of one equatorial oxygen in the h-bond leads to significant lengthening of its coordination bond in comparison to the rest of the three equatorial bonds (Table 2). This phenomenon is observed for the whole [Cu_2_(A)_4_(HA)_2_] group (Figure 3d) and the mean difference between the longest equatorial bond and the rest equatorial bonds is 0.03 Å. Only one exception is the compound of triphenylactetic acid (CSD refcode: ROLZUN [16]), in which the equatorial coordination bond involved in the h-bond has the second-shortest length (white stars in Figure 3d). This is a consequence of a large steric hindrance of ligands. Because four bridging triphenylacetate anions cannot rich the most favorable position in the inner coordination sphere, they form strongly asymmetrical bridges. 

**Figure 4:**
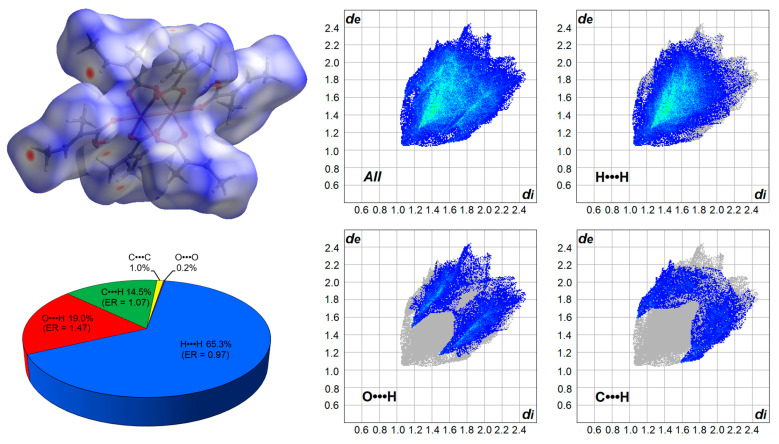
Hirshfeld surface (plotted over d_norm_), 2D fingerprint plots, and percentage distribution of the intermolecular contacts in the studied compound.

The crystal structure of the studied compound is stabilized by dispersive interactions and weak hydrogen bonds. The above discussed O-H•••O hydrogen bond is an intramolecular interaction; thus it does not influence molecular packing. The analysis of the Hirshfeld surface and the 2D fingerprint plots revealed that the greatest contribution in intermolecular contacts has dispersive H•••H interactions (Figure 4), which are formed between hydrogens of two methyl groups or methyl and methine groups. The next in turn of these are the weak hydrogen bonds C-H•••O and C-H•••C. Enrichment ratios for such three main interaction types are very close or higher than 1 (Figure 4), which indicates that they are favorable for the crystal net [40]. The ER value for C-H•••O is the largest, which means that these interactions play a slightly more important role in supramolecular assembling in comparison to the rest. 

### 3.2. IR Spectroscopy Analysis

**Figure 5:**
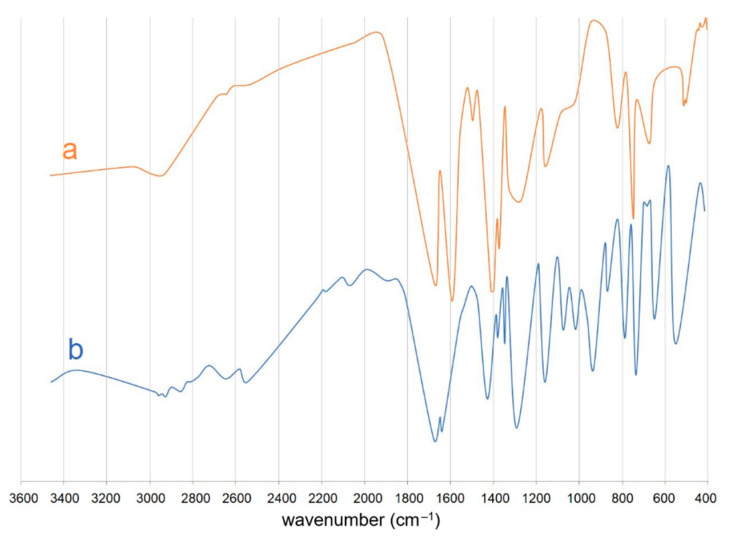
FT-IR spectra of [Cu_2_(tig)_4_(tigH)_2_] (**a**) and tiglic acid (**b**).

The spectrum of the studied compound contains absorption bands in the regions, which are characteristic of tiglic acid and its anion (Figure 5). For pure acid, the ν(C=O) vibrations are identified as the strong bands at 1678 and 1639 cm^−1^. In its spectrum, there are also ν(OH) and δ(OH) modes in the ranges 3300–2500 cm^−1^ and 1440–1380 cm^−1^, respectively. Additionally, bands of the stretching CH vibrations exist in the region 3300–2500 cm^−1^. Characteristic absorption modes for ν(C-O) appear at 1348 and 1293 cm^−1^. In the region 900–600 cm^−1^, bands of γ(CH) are observed. The spectrum of [Cu_2_(tig)_4_(tigH)_2_] is poorer than that of free acid. In comparison to tiglic acid, all bands are shifted to higher and lower frequencies as a result of the coordination process. The bands at 1677 cm^−1^, 1399 cm^−1^, 1372 cm^−1^, and 1269 cm^−1^ are assigned respectively to ν(C=O), δ(OH) and ν(C-O), and they are the evidence of the presence of coordinated acid molecules in the copper compound. In its spectrum, there are also bands characteristic of tiglate anions. The most important are two bands originating from stretching vibrations of the carboxylate group: ν_as_(COO) at 1591 cm^−1^ and ν_s_(COO) at 1496 cm^−1^. It proves that COO groups are bonded to copper(II) ions. In the spectrum of the copper compound, the absorption bands of γ(CH) exist at 824 cm^−1^, 749 cm^−1^, and 673 cm^−1^. 

### 3.3. Thermal Analysis

**Figure 6:**
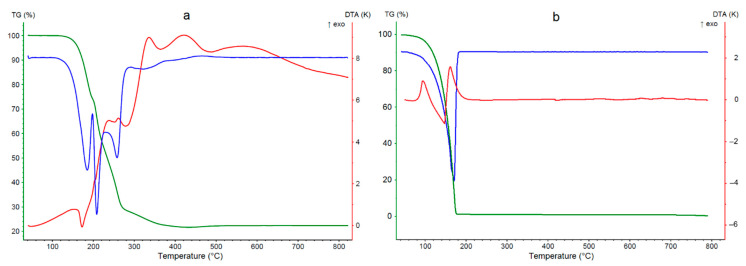
TG (green), DTG (blue), and DTA (red) curves for [Cu_2_(tig)_4_(tigH)_2_] (**a**) and tyglic acid (**b**).

The studied coordination compound is stable up to 120 °C (Figure 6a). The first step of decomposition takes place in the temperature range 120–200 °C and is associated with a loss of two molecules of tiglic acid (mass loss 26.7%, calculated 27.6%). This is an endothermic process with the maximum on the DTA curve at 175 °C. For comparison, thermal decomposition of free tiglic acid starts at 50 °C (Figure 6b), and this is an exothermic process (two peaks at 90 and 170 °C on the DTA curve). It proves that the formation of coordination bonds makes tiglic acid more thermally stable and changes the mechanism of decomposition (exothermic for free acid and endothermic for coordinating acid). The second decomposition step of [Cu_2_(tig)_4_(tigH)_2_] is the disintegration of tiglate anions (mass loss 51.4%, calculated 50.3%). It occurs directly after the decomposition of tiglic acids and ends at 415 °C. It is an exothermic process composed of several substages (peaks at 240, 260, 340, and 410 °C on DTA curve). The mass residue is 21.9%, which indicates that the final solid product is CuO (calculated 22.1%). 

**Figure 7:**
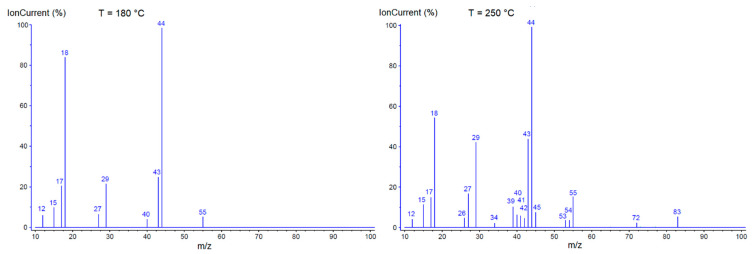
Mass spectra of volatile products from the thermal decomposition of [Cu_2_(tig)_4_(tigH)_2_] registered at 180 °C and 250 °C. The elaboration of the mass spectra involved subtracting the background spectrum and application of an automatic software correction for the carrier gas.

Mass spectra registered during the thermal analysis of [Cu_2_(tig)_4_(tigH)_2_] revealed the volatile products formed during decomposition (Figure 7). Major maxima for ion currents were observed at temperatures 180 °C (the first decomposition step) and 250 °C (the second decomposition step). The signals for m/z = 12, 17, 18, 44, 45, 46 correspond to C^+^, OH^+^, H_2_O^+^, CO_2_^+^, and they are connected with the combustion of organic ligands. Other signals (m/z = 15, 26, 27, 29, 34, 39, 40, 41, 42, 53, 54, 55, 72, and 83) are the result of fragmentation processes of ligands.

### 3.4. SQUID Magnetization Analysis

**Figure 8:**
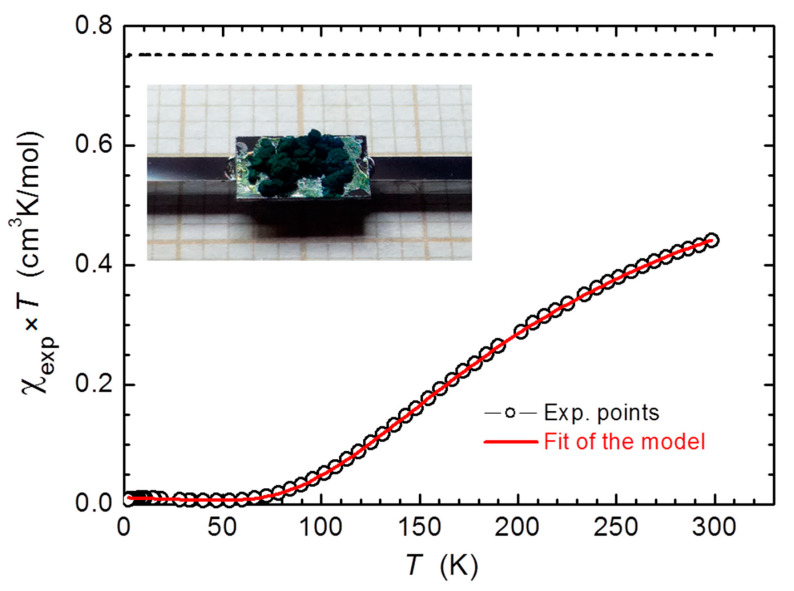
Experimental magnetic susceptibility *χ*_exp_ × *T* of [Cu_2_(tig)_4_(tigH)_2_] plot vs. temperature *T*. Experimental points are marked by black circles and the results of the modelling including T-dependent response of a Cu(II)–Cu(II) dimer (mid-to-high T range), a Curie paramagnetic-like contribution (very low temperatures) and T-independent diamagnetism of the host are depicted as the red solid line. The black dashed line denotes the Curie constant calculated for two noninteracting *S* = ½ spins. The inset: [Cu_2_(tig)_4_(tigH)_2_] powder specimen attached to the rectangular Si support plate and mounted onto a Si holder.

Figure 8 shows the temperature dependence of the molar magnetic susceptibility *χ*_exp_(*T*) of 3.4 ± 0.2 mg sample of [Cu_2_(tig)_4_(tigH)_2_] measured at *H* = 0.6 T and presented as *χ*_exp_ × *T* plot (black circles). A slow roll down of *χ*_exp_ × *T* on lowering *T* confirms that the Cu(II)–Cu(II) coupling is antiferromagnetic, which means that the low temperature ground state of this dimer is a nonmagnetic singlet. A relatively strong magnetic response at high *T*, above 60% of the expected signal of two noninteracting Cu(II) spins, indicates that the energy separating the ground singlet state from the excited triplet states, −2 *J*, is of the order of thermal energy at room temperature, *k*_B_*T* ≅ 25 meV ≅ 200 cm^−1^; *k*_B_ is the Boltzmann constant. This allows a substantial population of the lowest, the magnetically active *m*_S_ = −1 triplet state and so such a substantial magnetic response above some 150 K. The magnitude of the antiferromagnetic exchange coupling −2 *J* was estimated by fitting to the experimental results the model Bleaney–Bowers formulae describing the *T*-dependence of the magnetic susceptibility of two interacting *S* = ½ spins defined by the Hamiltonian H = −2 *JS*_1_*S*_2_ [41]. The Curie law contribution was also added to give an account of a paramagnetic-like increase of *χ*_exp_ at very low temperatures due to some structural defects [42] and a *T*-independent parameter describing the diamagnetism of the host structure, *χ*_dia_. In the performed modeling −2 *J*, *χ*_dia_, and two molar concentrations of Cu dimers and paramagnetic defects, *n*_dimer_ and *n*_para_, respectively, were the fitting parameters. The resulting fit to *χ*_exp_(*T*) is denoted in Figure 8 by the red solid line. It has been obtained for −2 *J* = 292.0(3) cm^−1^, *n*_para_ = 0.008, and *n*_dimer_ = 1.09. The magnitude of −2 *J* established for the studied compound matches almost perfectly the exchange integral found in copper acetate [Cu_2_(CH_3_COO)_4_(H_2_O)_2_]: 286 cm^−1^ [14], which is a consequence of similar electronic properties of tiglate and acetate anions (pKa = 4.96 [43] and 4.76 [44], respectively). If analogous dicopper compound [Cu_2_(CCl_3_COO)_4_(CCl_3_COOH)_2_] is composed of a stronger carboxylic acid (pKa for trichloroacetic acid is 0.66 [44]), −2 *J* value decreases to 240 cm^−1^ [45]. Given an experimental uncertainty of the absolute mass of the powder used to prepare the specimen (about 6%), *n*_dimer_ was found to be very close to the expected value of unity. Since *n*_para_ is close to zero the magnetic studies confirm a very high structural and chemical constitution of the synthesized material.

## 4. Conclusions

The reaction between copper(II) carbonate hydroxide and tiglic acid led to obtaining the dinuclear copper compound of the paddle-wheel structure composed of four syn–syn bridging tiglate anions and two monodentate tiglic acid molecules. The structural data supported by the bond valence theory proves that interaction between copper cations in [Cu_2_(tig)_4_(tigH)_2_] has a nonbonding character. The OH group of tiglic acid forms an intramolecular hydrogen bond with carboxylate oxygen of one tiglate anion. The involvement of one tiglate oxygen in h-bond leads to significant lengthening of its coordination bond with copper in comparison to the rest of the tiglate oxygens. The supramolecular structure of the studied compound is stabilized by H•••H dispersive interactions and weak C-H•••O and C-H•••C hydrogen bonds. The FT-IR spectrum contains bands corresponding to both tiglic acid and tiglate anion. The vibration modes of carboxylic and carboxylate groups are well distinguishable. Thermal analysis showed that tiglic acid molecules decompose before tiglate anions. The final product of decomposition is CuO. The magnetic measurements of the studied material indicate its very high structural and chemical quality and yield the antiferromagnetic configuration of Cu(II) ions, thus opening wide prospects of utilization in various fields as, e.g., biosensors, capacitors, transistors, or in data storage systems [46,47,48,49].

## Figures and Tables

**Table 1:** Crystal data and structure refinement details for [Cu_2_(tig)_4_(tigH)_2_].

Empirical formula	C_30_H_44_Cu_2_O_12_
Formula weight	723.73
Crystal system	Monoclinic
Space group	*P*2_1_/*c* (No. 14)
Temperature (K)	100.0(1)
X-ray wavelength (Å)	*λ*(Cu*Kα*) 1.54184
Unit cell dimensions	
a (Å)	9.2533(1)
b (Å)	17.4061(1)
c (Å)	10.2739(1)
α (°)	90
β (°)	95.113(1)
γ (°)	90
Volume (Å^3^)	1648.17(3)
Z	2
Calculated density (Mg/m^3^)	1.458
Absorption coefficient (mm^−1^)	2.101
*F(000)*	756
Crystal size (mm)	0.078 × 0.093 × 0.098
*θ* Range for data collection (°)	4.798 to 78.728
Index ranges	−11 ≤ h ≤ 11, −21 ≤ k ≤ 21, −13 ≤ l ≤ 12
Reflections collected/unique	33781/3438
R*_int_*	0.0252
Completeness (%)	100.0 (to *θ* = 67°)
Min. and max. transmission	0.50235 and 1.00000
Data/restraints/parameters	3438/0/206
Goodness-of-fit on *F*^2^	1.059
Final *R* indices [*I* > 2σ(*I*)]	*R*1 = 0.0247, *wR*2 = 0.0663
R indices (all data)	*R*1 = 0.0252, *wR*2 = 0.0666
Largest diff. peak and hole (e•Å^−3^)	0.345 and −0.359

**Table 2:** Selected structural data of the studied compound.

i—j	d_ij_ (Å)	*ν*_ij_ (v.u.)	i—j—k	α_ijk_ (°)	i—j—k	α_ijk_ (°)
Cu1—O1	1.9530(10)	0.448	O1—Cu1—O2^i^	169.39(4)	O2^i^—Cu1—O4^i^	88.42(4)
Cu1—O2^i^	1.9955(10)	0.399	O1—Cu1—O3	89.93(5)	O2^i^—Cu1—O5	91.76(4)
Cu1—O3	1.9515(10)	0.450	O1—Cu1—O4^i^	90.37(4)	O3—Cu1—O4^i^	169.61(4)
Cu1—O4^i^	1.9528(10)	0.448	O1—Cu1—O5	98.85(4)	O3—Cu1—O5	94.02(4)
Cu1—O5	2.1991(10)	0.230	O2^i^—Cu1—O3	89.38(4)	O4^i^—Cu1—O5	96.19(4)
Cu1•••Cu1^i^	2.5956(4)	–	–	–	–	–
D—H•••A	d(D—H) (Å)		d(H•••A) (Å)	d(D•••A) (Å)	<(DHA) (°)	Graph-Set
O6—H6O•••O2^i^	0.82		1.82	2.6345(14)	170.3	S(6)

The bond valences were calculated as *ν*_ij_ = exp*[(R_ij_-d_ij_)/b*] [34,35], where *R_ij_* is the bond-valence parameter for *i-j* bond (*R*_Cu-O_ = 1652 Å [36]) and *b* is the constant equaled 0.37 Å [37]. Symmetry transformations used to generate equivalent atoms: (i) −x + 1, −y + 1, −z + 1.

## Data Availability

The data presented in this study are available on request from the corresponding author.

## Supplementary Materials

The supplementary materials are available online at https://www.mdpi.com/article/10.3390/ma14092148/s1.
