# Supplementary material for: Structural, Spectroscopic, Thermal, and Magnetic Properties of a New Dinuclear Copper Coordination Compound with Tiglic Acid
Source: Materials (Basel). 2021 Apr 23;14(9):2148. doi: 10.3390/ma14092148 (PMC8122876; doi:10.3390/ma14092148)

checkCIF/PLATON report

Structure factors have been supplied for datablock(s) sun_cu_tygl

THIS REPORT IS FOR GUIDANCE ONLY. IF USED AS PART OF A REVIEW PROCEDURE FOR PUBLICATION, IT SHOULD NOT REPLACE THE EXPERTISE OF AN EXPERIENCED CRYSTALLOGRAPHIC REFEREE.

No syntax errors found.   CIF dictionary   Interpreting this report

Datablock: sun_cu_tygl

```
Bond precision:  C-C = 0.0020 A          Wavelength=1.54184

Cell:            a=9.2533(1)     b=17.4061(1)      c=10.2739(1)
                 alpha=90        beta=95.113(1)    gamma=90
Temperature:     100 K

                Calculated                 Reported
Volume          1648.17(3)                 1648.17(3)
Space group     P 21/c                     P 21/c
Hall group      -P 2ybc                    -P 2ybc
Moiety formula  C30 H44 Cu2 O12            C30 H44 Cu2 O12
Sum formula     C30 H44 Cu2 O12            C30 H44 Cu2 O12
Mr              723.75                     723.73
Dx,g cm-3       1.458                      1.458
Z               2                          2
Mu (mm-1)       2.101                      2.101
F000            756.0                      756.0
F000'           750.29
h,k,lmax        11,22,13                   11,21,13
Nref            3551                       3438
Tmin,Tmax       0.814,0.849                0.502,1.000
Tmin'           0.814

Correction method= # Reported T Limits: Tmin=0.502 Tmax=1.000
AbsCorr = MULTI-SCAN

Data completeness= 0.968          Theta(max)= 78.728

R(reflections)= 0.0247( 3354)     wR2(reflections)= 0.0666( 3438)

S = 1.059                 Npar= 206
```

The following ALERTS were generated. Each ALERT has the format
test-name_ALERT_alert-type_alert-level.
Click on the hyperlinks for more details of the test.

Alert level C

```
PLAT977_ALERT_2_C Check Negative Difference Density on H10C            -0.33 eA-3
```

Alert level G

```
PLAT007_ALERT_5_G Number of Unrefined Donor-H Atoms .............          1 Report
PLAT142_ALERT_4_G s.u. on b - Axis Small or Missing .............    0.00010 Ang.
PLAT143_ALERT_4_G s.u. on c - Axis Small or Missing .............    0.00010 Ang.
PLAT153_ALERT_1_G The s.u.’s on the Cell Axes   are Equal ..(Note)     0.0001 Ang.
PLAT380_ALERT_4_G Incorrectly? Oriented X(sp2)-Methyl Moiety .....         C4 Check
PLAT794_ALERT_5_G Tentative Bond Valency for Cu1       (II)      .       2.10 Info
PLAT912_ALERT_4_G Missing # of FCF Reflections Above STh/L=  0.600        108 Note
PLAT978_ALERT_2_G Number C-C Bonds with Positive Residual Density.         10 Info
```

```
   0 ALERT level A = Most likely a serious problem - resolve or explain
   0 ALERT level B = A potentially serious problem, consider carefully
   1 ALERT level C = Check. Ensure it is not caused by an omission or oversight
   8 ALERT level G = General information/check it is not something unexpected

   1 ALERT type 1 CIF construction/syntax error, inconsistent or missing data
   2 ALERT type 2 Indicator that the structure model may be wrong or deficient
   0 ALERT type 3 Indicator that the structure quality may be low
   4 ALERT type 4 Improvement, methodology, query or suggestion
   2 ALERT type 5 Informative message, check
```

It is advisable to attempt to resolve as many as possible of the alerts in all categories. Often the minor alerts point to easily fixed oversights, errors and omissions in your CIF or refinement strategy, so attention to these fine details can be worthwhile. In order to resolve some of the more serious problems it may be necessary to carry out additional measurements or structure refinements. However, the purpose of your study may justify the reported deviations and the more serious of these should normally be commented upon in the discussion or experimental section of a paper or in the "special_details" fields of the CIF. checkCIF was carefully designed to identify outliers and unusual parameters, but every test has its limitations and alerts that are not important in a particular case may appear. Conversely, the absence of alerts does not guarantee there are no aspects of the results needing attention. It is up to the individual to critically assess their own results and, if necessary, seek expert advice.

Publication of your CIF in IUCr journals

A basic structural check has been run on your CIF. These basic checks will be run on all CIFs submitted for publication in IUCr journals (*Acta Crystallographica*, *Journal of Applied Crystallography*, *Journal of Synchrotron Radiation*); however, if you intend to submit to *Acta Crystallographica Section C* or *E* or *IUCrData*, you should make sure that full publication checks are run on the final version of your CIF prior to submission.

Publication of your CIF in other journals

Please refer to the *Notes for Authors* of the relevant journal for any special instructions relating to CIF submission.

PLATON version of 22/03/2021; check.def file version of 19/03/2021

Datablock sun_cu_tygl - ellipsoid plot

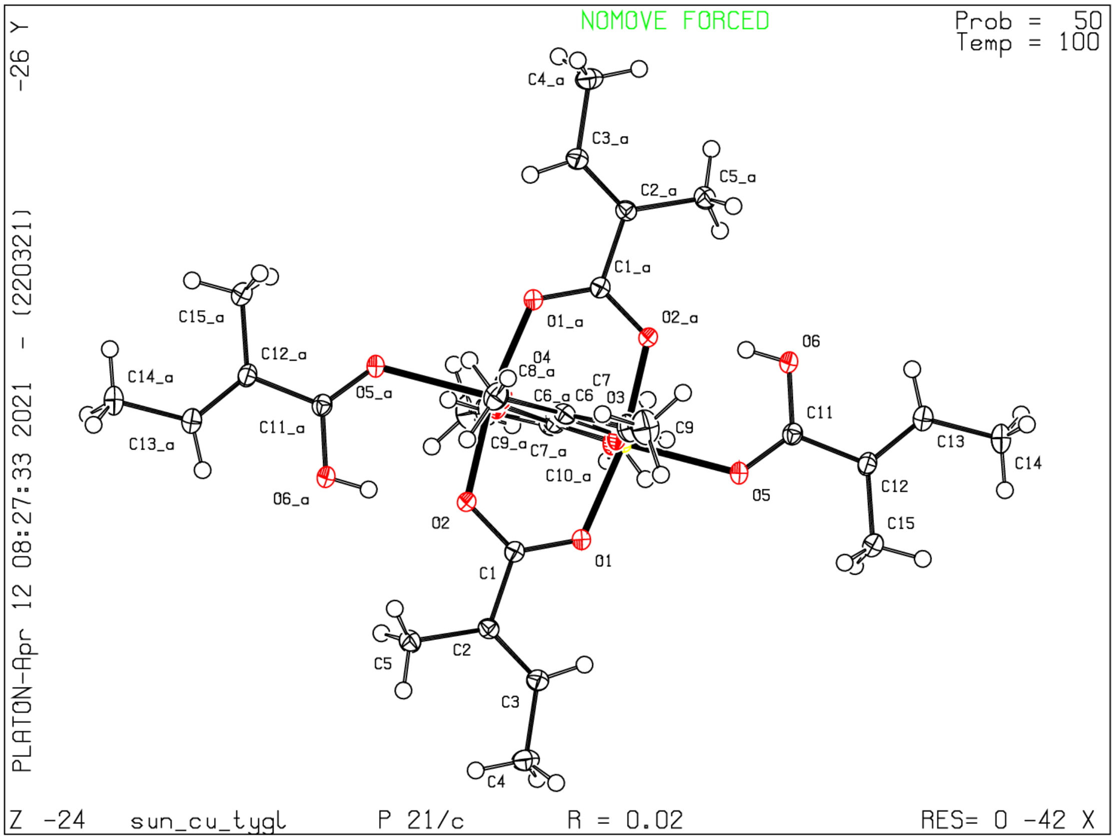

Supplement: Supplementary file 1 [file materials-14-02148-s001.zip › checkcif.pdf]
